# Criterion-Referenced Assessment of Intelligence as Adaptation to the Environment: Is It Possible, Plausible, or Practical?

**DOI:** 10.3390/jintelligence10030057

**Published:** 2022-08-16

**Authors:** Robert J. Sternberg, Aakash Chowkase, Fabio Andres Parra-Martinez, Jenna Landy

**Affiliations:** 1 Department of Psychology, Cornell University, Ithaca, NY 14853, USA; 2 Department of Educational Studies, Purdue University, West Lafayette, IN 47906, USA; 3 Department of Education Reform, University of Arkansas, Fayetteville, AR 72701, USA

**Keywords:** criterion-referenced testing, norm-referenced testing, intelligence, adaptive intelligence, achievement

## Abstract

Criterion-referenced testing is usually applied to the assessment of achievement. In this article, we suggest how it can also be applied to the assessment of adaptive intelligence, that is, intelligence as adaptation to the environment. In the era of the Anthropocene, we argue that adaptive intelligence is what is most important not only for individual success, but also for success in terms of preservation of the world as we know it. We define criterion-referenced testing and compare it to norm-referenced testing. We then discuss two kinds of scoring of criterion-referenced testing, namely, with respect to external criteria and with respect to internal (theory-based) criteria. We then discuss past research on intelligence that could be viewed as criterion-referenced. Finally, we suggest how criterion-referencing could be applied to the assessment of adaptive intelligence.

## 1. Introduction: Intelligence in the Era of the Anthropocene

In the era of the Anthropocene, what matters most to the world is, arguably, not how well an individual performs intelligently in comparison to that individual’s norm-referenced group, but rather, how well an individual performs intelligently in terms of adaptation that helps to preserve and enhance the world. The world faces huge challenges—for example, climate change, pollution, massive income inequality, pandemics, weapons of all kinds—and performing well in terms of others’ performance is not enough. For example, contributing less to climate change than one’s neighbors may still leave one contributing grossly and negligently to climate change. Polluting less than one’s neighbors still may leave one as a polluter who is contributing to fouled air. Corporations, especially, seem susceptible to the notion that, well, they pollute, but they pollute less than a competitor. We need to set adaptively intelligent standards that are more rigorous than just that one person (or group entity) is doing better or worse than another. Criterion-referenced assessment evaluates performance not in terms of one’s peers, but in terms of meaningful criteria that an individual or group should strive to fulfill.

There are two broad categories of assessment (Hambleton 1980; Hambleton and Novick 1973; Popham 2014): norm-referenced and criterion-referenced. With norm-referenced assessment, the basis for evaluating individuals is with respect to how they compare with other individuals taking the same test or an equivalent form of the test. For example, the current version of the Stanford-Binet Intelligence Scales (5th ed.—Roid 2003) and of the Wechsler Intelligence Scale for Children (5th ed.—Wechsler 2014) both use norm-referencing. With this procedure, norms, or relative expectations for performance, are established by comparing the performances of individuals to each other. Standardized scores (in this case, IQs) are then assigned based on the relative performances. These scores are typically derived from percentile equivalents.

The purpose of this article is to present an approach to criterion-referenced assessment of intelligence as adaptation, also called *adaptive intelligence* (Sternberg 2021b). Intelligence is almost always measured in a norm-referenced way because, historically, it has not been clear what the criterion or criteria would be with respect to which assessments of intelligence could be criterion-referenced. We argue in this conceptual article that for adaptive intelligence there are such criteria, namely, world-level problems, identified by the United Nations and other prestigious international bodies, that must be solved for the world to remain adaptable to. That is, unless these problems are solved, there is a serious risk of losing a planet suitable for, or even habitable by, human life.

Whereas norm-referenced assessments (NRA) score interpretations seek to situate the test-taker’s score in an ordered continuum relative to other test-takers’ performance, criterion-referenced assessment (CRA) is a strategy to interpret test scores based on the proficiency of a person as compared to a criterion or standard of performance that is independent of other test takers (Glaser 1963; Popham and Husek 1969). Thus, it does not matter who else takes the given test; the criterion-referenced score is with reference to an external criterion that is independent of the population being tested.

CRA test scores provide meaningful interpretable values about the performance of a test-taker within the examined criteria. The criterion is typically a set of well-defined learning objectives or competencies (Hambleton 1980). CRA scores provide explicit information about tasks the test-taker can or cannot do, given a domain of performance, or how much the test-taker knows of a given field of knowledge. Criteria or standards for performance are externally determined. Test developers and stakeholders make decisions about the criteria and levels of performance according to the situation in which the knowledge or skills assessed will be applied.

The definition and distinction between criterion-referenced and norm-referenced measures lie in the interpretation of test scores rather than in the nature of the test (Millman 1974; Popham 2014). Some objectives of criterion-referenced test score interpretations include:

to describe, clarify, and communicate requirements; to contextualize and fine-tune expectations; to facilitate the substantiation of judgments; to safeguard against subjectivity and bias; to ensure fairness; and to provide a defensible framework for assessing.

(Scarino 2005, p. 9)

Criterion-referenced interpretations offer multiple uses for educational and other purposes. They allow professionals to “monitor individual progress in objectives-based instructional programs, to diagnose learning deficiencies, to evaluate educational and social action programs, and to assess competencies on various certification and licensing examinations” (Hambleton et al. 1978, p. 2). They allow educators to know what a test-taker can do in terms of achieved levels of mastery, rather than merely in relative terms to what other individuals can do.

Advantages of CRA interpretations over NRA interpretations, according to Hambleton (1993), include that CRA: (a) specifies clear performance objectives and outcomes established by experts for various content areas; (b) items are established with the clear goal of possessing content validity for whatever outcomes are being measured; (c) allows for assessment of mastery levels in terms of important content, rather than relative performance; (d) forces test-constructors to establish criterion-related standards of performance; (e) shows specific areas of strength and weakness relative to the established criteria and; (f) specifically demonstrates what instruction needs to be done to achieve mastery. According to O’Donovan et al. (2001), additional advantages of CRA measures include providing objective guidelines to improve student work, facilitating feedback, and improving standards and grading consistency. 

Clearly, the criterion-referencing is only as good as the experts setting and applying the criteria and scaling them for the test (Burton 2006; Rovinelli and Hambleton 1976). It is important, therefore, that those setting up the criterion referencing are experts in the given field of endeavor.

## 2. Levels of Criterion-Referenced Testing

Criterion-referenced testing can be viewed in much the same way that sampling of populations of persons can be viewed. In the latter case, the source of variation is persons; in the former case, it is items measuring various kinds of content and mental processes.

## 3. Criterion-Referenced with Respect to What?

Criterion-referencing of achievement tests is typically done with respect to an external criterion, such as one’s grade level in spelling, vocabulary, or arithmetic. In this case, the criterion is the expectation of a particular level of knowledge by a certain school grade. In some cases, the criterion may be more straightforward. For example, if one needs to memorize a particular text, such as a poem, one can evaluate learning in terms of the percentage of the text that is memorized. Percentage grades in schools are supposed to be criterion-referenced, although they often do not really refer only to the percentage of material of the course that has been learned.

Criterion-referencing also can be done with respect to an internal criterion, such as the specifications of a particular theory. Often, whether or not there is an explicit theory, there is an implicit theory being used without users even recognizing it. For example, suppose a reading test provides a score for percentage comprehension of material and reading speed. In that case, the implicit theory is that reading comprehension and reading speed are what matter in assessing reading. An alternative implicit theory might also assess grade (or some other) level of vocabulary, asserting that vocabulary, too, is an important measure of reading skill, as one cannot read adequately if one does not understand all or at least most of the words.

The main way in which a form of criterion referencing has been done in the intelligence literature is through the use of mental age (MA), or expectations about what level of mental performance can be expected of an individual of a given chronological age. The problems with the mental age construct are by now well known (see, e.g., Sternberg 2020). First, the mental age construct begins to break down around the chronological age of 16: Mental growth slows down noticeably after about 16 years of age. Second, mental growth does not show the pattern of continuous development that the mental-age concept would imply (any more than physical growth shows a continuous pattern of development). Third, it is unclear that there is any one mental age that well characterizes an individual; individuals show profiles of strength and weakness that the MA construct does not well capture. Very few intelligence tests use the mental age construct anymore. Rather, IQ is computed in terms of standard-score and percentile measurements, which are norm-referenced.

Note that the goal of criterion-referenced testing is not to be more “accurate” or necessarily more predictively valid than norm-referenced testing. Indeed, often the exact same test can be scored in a norm-referenced or criterion-referenced way. Instead, the purpose is to understand a test score with respect to an external criterion of consequence, rather than with respect to the performance of a normative reference group. One kind of scoring is not intrinsically “better” than the other. Rather, each kind of scoring has a different purpose.

## 4. External Criterion-Referencing

The selection of items for external criterion referencing can be accomplished in a variety of ways.

### 4.1. Universe of Relevant Knowledge

A first option is that the test constructor tests the entire universe of content that is of interest for a particular purpose. For example, if an individual memorizes a fixed set of religious prayers or a religious text, it is straightforward to test the entire universe relevant for the purpose. Of course, the domain need not be religious. The universe could be the complete set of German pronouns in all grammatical cases, a poem, or the entire periodic table of elements. In these cases, one does not have to worry about the sampling of content.

### 4.2. “Most Important” Knowledge of a Domain

A second option is to test knowledge designated by experts as most important for test-takers to know. For example, it would be practically impossible to test anyone’s knowledge of the meanings of all words in English, French, or any other language. Rather, one could decide on a basis for designating certain words as “important”. These might be words that occur in texts with high frequency, or they might be words that content experts believe all college students (or individuals in some other population) should know. In a given discipline, similarly, there might be certain terms that stand out from others in terms of either their frequency of use, or their judged importance to the field.

### 4.3. “Representative” Knowledge of a Domain

A third option is to test knowledge that content-domain experts deem to be representative of what one needs to know. Achievement tests generally measure the representation of knowledge. They cannot measure the entirety of the subject matter, so they cover material that is representative of the domain. For example, in psychology, a test might sample biological, clinical, cognitive, developmental, and social-personality psychology.

### 4.4. “Randomly Sampled” Knowledge within a Domain

Finally, in a large domain of knowledge, it may be easiest simply to randomly sample knowledge to ascertain what proportion of knowledge, or what kinds of knowledge, the test-taker possesses. For example, if the test-taker has memorized a book or a speech or script, they can be asked to repeat material from one or more randomly chosen portions of the learned material. 

## 5. Internal Criterion-Referencing: Attempts to Apply Criterion-Referencing to the Testing of Intelligence

Tests of intelligence can be criterion-referenced in a variety of ways. One would be with respect to grade or level of vocabulary, or some other external knowledge base (for measuring crystallized intelligence—Carroll 1993; Cattell 1971). Or, for fluid intelligence, an internal criterion-referencing might be used, by linking, for example, performance on the test to aspects of a theory of intelligence, such as requirements of a given test item in terms of storage capacity or central-executive processing (e.g., Primi 2014). 

There have been various attempts to study intelligence in a criterion-referenced way, although they generally have not been referred to in that way. Part of the reaction against psychometric methodology in the 1970s was a reaction against the use of wholly norm-referenced assessments, relying, as these assessments did, on norms of individual differences (e.g., Sternberg 1977). Sternberg (1977, 1983) and Hunt (Hunt et al. 1973, 1975), as well as Jensen (1982a, 1982b), were all seeking to understand intelligence not by deriving factors from patterns of individual differences, but rather from patterns of item reaction-time differences. The difference was that, with their methods, ability scores would be characterized not by scores on allegedly underlying ability factors based on individual differences, but rather by scores derived from component analyses of reaction times (Sternberg 1985). Other cognitive psychologists were using related methods for studying other kinds of cognitive tasks, such as sentence-picture comparisons (Clark and Chase 1972), matrix solution (Carpenter et al. 1990), and mental rotation (Shepard and Metzler 1971). 

The idea in this kind of componential analysis (Sternberg 1983), or cognitive-correlates analysis (Hunt et al. 1975), was to isolate the components of information processing and then specify the actual amount of time the components consumed during that information processing. The criterion referencing, then, was in terms of the amount of time spent per process, typically expressed in milliseconds. It was further possible to discern the strategies used and whether those strategies were optimal. What made the effort significant was that the tasks were not ones that were chosen *post hoc* because, for example, they were viewed as school-relevant and, at the same time, showed age differences in success rates (Binet and Simon 1916), but rather because they were theorized to be privileged, in the sense that they measured what were alleged to be the true information-processing bases of intelligence. 

Spearman (1923) made the processes of analogical reasoning—what he called apprehension of experience, eduction of relations, and eduction of correlates—the centerpiece of his cognitive theory of intelligence, as did Sternberg (1977), using different terminology and an expanded set of processes. Apprehension of experience was encoding of analogy terms; eduction of relations was the inference of how two analogy terms were related; and eduction of correlates was the application of the inferred rule to a new domain. 

Hunt (1980) suggested that the speed of lexical retrieval was a key to verbal intelligence. And Carpenter et al. (1990) studied the Raven Progressive Matrices (Raven 1938; Raven 1986), which have sometimes been considered to be a relatively pure measure of general intelligence. More recent work has used tests of working memory as bases for understanding general intelligence (Conway and Kovacs 2013, 2020; Ellingsen and Engle 2020; Engle 2018; Engle and Kane 2004). Primi (2014), mentioned above, used criterion-referencing for matrix-type problems.

## 6. Adaptive Intelligence

Sternberg (2019a, 2021a, 2021b) proposed a concept of adaptive intelligence, according to which intelligence is adaptive when it is used to adapt to the world, in particular, to help to make the world a better place by achieving a broad, common good. In our view, such intelligence is especially important in the era of the “Anthropocene”, in which humans are not only changing the nature of the Earth, but also the living conditions for the diverse species inhabiting the Earth. 

Sternberg (2021a) argued that conventional intelligence is often being used for indifferent or even destructive purposes, which has created short-term gains but long-term losses, such as global climate change, air and water pollution, violence by guns and other weapons including weapons of mass destruction, and spread of pandemics. For example, very clever campaigns have been undertaken, usually for ideological or political purposes, to discourage measures to protect people from COVID-19, with the result that people have died of the novel coronavirus who might not otherwise have died (Robins-Early 2022). As of the day on which we are writing these words, 17 June 2022, more than 6.3 million people have died of COVID-19 and there have been over 538 million cases (https://www.google.com/search?client=firefox-b-1-d&q=how+many+people+have+died+of+covid-19+worldwide, accessed on 17 June 2022). Many of the deaths were unnecessary and could have been prevented (Amin et al. 2022). People may be adept at solving the abstract kinds of problems that appear on intelligence tests, but often seem not to use this intelligence in a way that is adaptive for humans, or other species either (Sternberg 2021a).

Whereas tests of general intelligence are almost all norm-referenced, there is a basis for criterion-referencing tests of adaptive intelligence. Sternberg’s (2021a) view, at least, is that people have a responsibility for the perpetuation of their species, as well as species that depend on humans in this age of the Anthropocene. This is what, biologically, natural selection and adaptive competence are about—reproduction of a gene pool. In this way, others can enjoy the opportunities one has had, oneself, in life. Criterion-referencing of adaptive intelligence would be in terms of the quality of responses to problems addressing the adaptive needs of humans for the biological imperative of the continuation of their own species. How might one decide on the criteria and how might one evaluate responses, based on those problems that are seen as relevant to humanity?

According to the theory of adaptive intelligence (Sternberg 2021a), one could evaluate responses in terms of how they help to promote the common good, by balancing the interests of all relevant parties to a decision or solution of a problem in the long- as well as the short-term, through the infusion of ethical values. Experts would rate responses in terms of these particular aspects, which derive from a balance theory of wisdom (Sternberg 1998, 2019b).

Adaptive intelligence (Sternberg 2021b) is different from practical intelligence (Sternberg et al. 2000), a related but distinct concept. Practical intelligence is used to advance oneself in life—to adapt to, shape, and select environments to achieve whatever it is that one wants to achieve in life. Adaptive intelligence is also practical, but it is oriented toward a common good—toward making the world a better place. Adaptive intelligence requires practical intelligence, but one could be practically intelligent without being adaptively intelligent, as are many highly “successful people” in society who achieve their success at the expense, or even at the cost, of the lives of others.

People’s ability to act for a common good is shaped and constrained by their individual circumstances. Individuals with high levels of material and other resources generally will be in a better position to work toward a common good than those with limited resources who can barely eke out a living. Whether the well-off actually will seek a common good will depend, in large part, on whether they are willing to give back as they have gotten, or rather, will see that what they have is never, ever enough, so that they seek only, or largely, to maximize their own individual gains. Yet, even subsistence farmers have something to offer to the world, such as food, livestock, or whatever else they have to offer. For example, research indicates individuals from low-income groups in collectivistic societies tend to be more empathically accurate in judging the emotions of other people (Kraus et al. 2010), are more attuned to others’ distress (Stellar et al. 2012), and act in a more prosocial manner because of a greater commitment to egalitarian values and feelings of compassion for others (Piff et al. 2010), compared with their high-income counterparts. On the contrary, individuals from upper-class backgrounds have the tendency to behave more unethically than their low-class counterparts, in part, accounted for by their more favorable attitudes toward greed (Piff et al. 2012). Similarly, the adaptive knowledge of indigenous communities around the globe has been well-documented (Stanistreet 2021; Tom et al. 2019). These communities can be seen as exemplars of deployment of adaptive intelligence based on “traditional ecological knowledge, farmer knowledge, and other forms of ecological knowledge [that] have served their populations for generations by facilitating thoughtful and deliberate human-environmental interactions leading to what is broadly referred to as environmental sustainability” (Tom et al. 2019, p. 12). Such endeavors not only satisfy the condition of adaptability but also the notion of common good, as indigenous efforts often result in group and global benefits. 

What is good for any given individual is not necessarily good for the organization, and vice versa (Molenaar 2004). For example, a business may lay off individuals because of an economic downturn; the business is able to survive, but the individual is out of a job. More generally, individual instances of seeking a common good do not and generally cannot benefit every individual every time. But when an organization needs to take actions that are disadvantageous to given individuals, it still can seek to help those who are harmed. It can do this in the instance given above, for example, by providing the discharged individual with severance, furnishing help to the individual in finding another job, and possibly in contributing to the individual’s retraining for another job. In almost any instance, some compensation can be achieved. The larger problem, we suggest, is when organizations are “heartless”, seeking only their own advantage, or that of their most powerful members, and viewing the rest as commodities who are disposable when the time comes. Acting in the common interest, and with heart, is possible anywhere and transcends cultural differences. It is a matter of humanity, not culture.

One could argue that what we call “adaptive intelligence” might better be called “adaptive competence”. Indeed, McClelland (1973) argued that society should test for “competence” rather than “intelligence”. On this view, one could argue that we have redefined intelligence toward a meaning it was never intended to have. We disagree with this view, however. On the contrary, the early thought leaders in the field of intelligence defined intelligence in terms of adaptation to the environment (e.g., Binet and Simon 1916; Thorndike 1921; Wechsler 1940). It is hard to see how many intelligence tests of today measure anything close to adaptation to the environment. Rather, they often are narrow measures of a subset of cognitive skills, such as some of those needed for academic success. IQ predicts many criteria (Sackett et al. 2020), but the criteria are almost all measures of short-term individual culturally sanctioned success. We know from the paradox of the tragedy of the commons that short-term individual successes can result in long-term collective disasters. Global climate change, air and water pollution, and much of the violence in the world, stem from what individuals or cultural groups view as culturally sanctioned short-term individual successes. The mistake, we believe, that much of the field of intelligence has made, is to fail to realize that the stakes today are such that there is little future for a world that takes such a narrow, individualistic, and ultimately egocentric view of “success”. On the random day we are writing (24 July 2022), heat records are being broken throughout the world (Patel 2022). More than 90 million people in the US are under extreme heat warnings (Elamroussi 2022). On this day, the highest recorded temperature is 134 degrees F. (56.7 °C). 

If we view intelligence only in terms of the usual short-term individual criteria, we would argue that humanity does not have much of a future on this world (see also Levi 2022; Ord 2020; Sternberg 2021b). We suggest instead that what we call “adaptive intelligence” is very much intelligence. Certainly, if humans commit species suicide, some future advanced species will consider them not only as incompetent, but as adaptively unintelligent in the extreme, whatever awards their successful members may have acquired, or however much money their most financially successful members may have made. From our point of view, the term “intelligence” has been co-opted in terms of cognitive skills that, while important to the life of an individual, have proved to be sadly inadequate to preserving humanity. When we think of *intelligence*, we should be thinking not merely of what is measured by academic tests of intelligence, but of what is needed, literally, to save humanity from itself. We should think not merely in terms of an “intelligence” that preserves and enhances the privileges of those who already have most benefited from current sociocultural systems, but also and more importantly, think in terms of what benefits the future of humanity and other species.

## 7. Priorities of Global Importance

To approach the challenge of developing a criterion-referenced method for testing adaptive intelligence, it is vital to establish the universe of topics to be assessed. As uses of adaptive intelligence seek to improve the world and work toward a common good, it stands to reason that assessed topics must be pressing and relevant priorities in the world today, and in the world of the future. Although there is no established consensus on the most pressing priorities in today’s world, the United Nations (UN) has provided and has continually updated a list of 17 sustainable development goals (United Nations Department of Economic and Social Affairs 2015), and a list of 23 global issues (United Nations n.d.a). By identifying main keywords relating to these 40 global priorities using an online keyword-identifying tool for research, and then searching for these keywords in major news outlets, substantial agreement on the most important topics is clearly identifiable. 

We surveyed eight major news outlets, including six general news outlets (*The New York Times*, *The Washington Post*, *The Los Angeles Times*, *The Miami Herald*, *The Chicago Tribune*, and *The Boston Globe*), one major scientific publication outlet (*Journal Storage—JSTOR*), and one major policy publication outlet (*Foreign Policy*). They were surveyed in May 2022 for keywords pertaining to the 40 identified priorities (De Vise 2011). The idea was to determine what priorities were consensual, beyond their listing by the U.N.

A major limitation of this selection is that many of the outlets (including all six of the general news outlets) are based in the United States, and therefore they may not provide a completely unbiased and accurate representation of global priorities. (The U.N., of course, is a broadly based international organization, but with headquarters in New York City.) Nevertheless, exploration of the selected news outlets revealed strong agreement among the news outlets, in terms of both “top ten priority” results (i.e., of the 40 priorities, this priority was among the ten that produced the most keyword search hits on the relevant websites), and “top three priority” results (i.e., of the 40 priorities, this priority was among the three that produced the most keyword search hits on the relevant websites). The five priorities of international relevance most agreed upon by these outlets were as follows: 

(1) *Health* or *Good Health and Wellbeing* was a “top ten priority” on all eight websites, and a “top three priority” on seven of the eight websites. On the UN website, *health* as a global priority pertains to the priorities of the World Health Organization (WHO), which mainly lists disease classification and treatment, improving suboptimal health conditions, and subsets of the global population susceptible to these diseases and conditions (United Nations n.d.b). At present, responding to and controlling the COVID-19 pandemic is a WHO top priority; this is in addition to the priorities settled on at the WHO’s establishment in 1948, which included malaria, venereal disease, nutrition and malnutrition, and the effects of environmental pollution (World Health Organization n.d.).

(2) *Children* or *Youth* was a “top ten priority” on seven of the eight websites, and a “top three priority” on four of the eight websites. On the UN website, *children* as a global priority pertains to topics addressed by the United Nations Children’s Fund (UNICEF), established by the UN in 1953 (United Nations n.d.c). The UN has established the Declaration of Rights of the Child, which defends “children’s rights to protection, education, health care, shelter, and good nutrition” (United Nations General Assembly 1959). UNICEF’s guiding principles are outlined in the Convention of Rights of the Child, a human rights treaty proclaiming childhood rights (up to age 18), such as special care, legal protection, freedom of thought, development in a loving family environment, and preservation of identity (i.e., family identity, cultural identity); the Convention also establishes that governments have an obligation to intervene in illicit child trade and labor (United Nations General Assembly 1989). The UN outlines the rights of youth (ages 15–24) in similar terms as those rights of children, adding a priority of just access to the job market, political participation, and economic growth, with a focus on equal access for marginalized populations (United Nations n.d.d).

(3) *Food* was a “top ten priority” on seven of the eight websites, and a “top three priority” on three of the eight websites. With factors such as high costs of food, increasing global food demand, and poor harvesting seasons, global hunger is on a steady rise. The UN has warned that the world is not on track to reach its goal of no global hunger by 2030; in fact, food security and nutrition are likely to worsen in the coming years (United Nations n.d.e). Beyond hunger, the UN has also focused on working toward healthier diets, eliminating malnutrition, developing sustainable and productive food production systems, and improving trade in world agriculture (United Nations n.d.e).

(4) *Water* was a “top ten priority” on seven of the eight websites, and a “top three priority” on two of the eight websites. The WHO and UNICEF have estimated that 2.2 billion people worldwide do not have access to safe drinking water (United Nations n.d.f). Beyond drinking water, the UN connects access to water to energy and food production, healthy environments and ecosystems, human health and survival, droughts and floods, hygiene and sanitary living conditions, and global climate change. The UN aims to improve access to clean water in poor countries, where water contamination contributes to poverty, disease, and child mortality (United Nations n.d.f).

Finally, (5) *Population* was a “top ten priority” on six of the eight websites, and a “top three priority” on one of the eight websites. The UN describes the global priority of *population* as pertaining to priorities stemming from the rapidly increasing global population, estimated to hit nearly 10 billion in 2050, and over 11 billion in 2100 (United Nations n.d.g). The UN has identified several reasons for population growth, including changes in fertility rates, longer lifespan, increased survivorship through reproductive age, and urbanization trends (United Nations n.d.g). A spiking population poses a major threat to future generations; specifically, their access to food and other limited resources.

Our argument, simply, is that the ability to reflect upon, analyze, and pose potential resolutions to problems such as these is more important to adaptivity than the ability to remember obscure vocabulary words or solve problem such as number- or letter- series problems, that are different in kind, scope, magnitude, and importance from real-world problems. The current intellectual power structure has been chosen in large part, through academic selection mechanisms, to excel in solution of relatively inconsequential problems, whereas the world needs people who excel in the solution of consequential ones. But any power structure tends to value whatever it is that put it into a position of power relative to those not in power (Sternberg 1997), so that change is hard to achieve. Whether the standard is general intelligence, socioeconomic status, a designated religion, a designated socially defined sex or race, or whatever, people are perfectly capable of convincing themselves that whatever attribute distinguishes them from others is an attribute that any among the “select” necessarily should have.

## 8. A Test to Measure People’s Adaptive Intelligence to Solve Priorities of Global Importance

The next step in developing a criterion-referenced test of adaptive intelligence consists of defining the characteristics of such a test. Ideally, a collaborative effort among measurement experts, educators, and content experts would be desirable to identify key areas of performance, and to differentiate levels of performance within the problem space of identified global issues. It is through this consensus that a criterion-referenced test could adequately provide evidence of the behaviors and abilities that people with adaptive intelligence can bring to bear about the pressing issues of the world, and how to solve them. Following Hambleton (1993) and Popham and Husek (1969), in terms of seeking advantages of criterion-referencing, four main characteristics and advantages of an adaptive-intelligence criterion-referenced test might be as follows.

### 8.1. Specific and Clear Standards

A test of adaptive intelligence would present a comprehensive listing and description of universally pressing issues affecting our world and calling for action. Overall, the proposed standards will emphasize the necessity for consistency and prioritization of global problems across different contexts and settings. For example, test developers could use insights from experts in social policy, human rights, environmental protection, and economic sustainability, among other fields, to identify the universe of relevant knowledge that is most important and representative. Test developers would establish the content, purpose, and design of the test. The test instructions would provide clear and specific information about the content and skills being evaluated. With overarching objectives in mind, the test developers then would design problem items and tasks that elicit adaptive intelligence, for instance, in the framework of sustainable development goals (United Nations n.d.a). 

A specific example of an item is presented later in this article, but a reasonable standard for inclusion of an item as criterion-referenced would be that the problem: (a) represents a major world problem as identified by the UN, or a comparably prestigious international body with recognized expertise; (b) is one that affects the future of humanity collectively; (c) is one about which any reasonably well-informed citizen of the world should be at least somewhat aware of and; (d) has multiple potential solutions of varying merit and varying risk levels. 

### 8.2. Established Levels of Performance 

Test developers also use standards to indicate the progression of performance levels of knowledge and ability to solve global issues. Such levels of performance are articulated cohesively and coherently following logical sequences. The test would include items that measure proposed behaviors according to each expected level of performance. Therefore, items must be relatively unambiguous indicators of a test-taker’s level of knowledge and ability to use that knowledge to identify and contribution to the enactment of potential solutions to the most pressing issues we have outlined. There would need to be consensus in the interpretation of test-taker scores across the different levels of performance measured by the test.

For a test of intelligence as adaptation, solutions could be evaluated in terms of the theory of adaptive intelligence (Sternberg 2021b). With established progressing levels of performance, learners’ scores would indicate attributes showing: (a) how creative they are—are they novel and potentially useful (will they contribute to solving the problem)?; (b) how analytically strong they are—are they logical, coherent, representing correct use of data?; (c) how practically strong they are—are they sensible, commonsensical, practical, plausible, capable of implementation?; (d) how wise they are—do they help to promote a common good by balancing one’s own, others’, and higher order interests, over the long- as well as the short-term, through the infusion of positive ethical values? Ultimately, the question is, how far will they go in terms of actually solving the given world problem?

### 8.3. Evidence for Decision Making 

A test of adaptive intelligence would show specific areas of strength and weakness relative to established criteria in test-takers’ performance. As a diagnostic tool, the test would facilitate decision-making by indicating the extent to which a person’s knowledge and ability to use that knowledge are suited for embarking on problem-solving for major world challenges. For example, this approach is useful for organizations and stakeholders to identify individuals with the ability and knowledge to serve as potential to lead, or join teams of positive change agents. 

A criterion-referenced test of intelligence as adaptation would reveal specific areas of strength and weakness in terms of understanding world-level problems that everyone who is a citizen of the world needs to understand. Those areas in which individuals, especially those of high school or college age, show weaknesses, ought to be areas in which the individuals improve their knowledge and understanding so that they can contribute as active concerned citizens, and ethical leaders, to the betterment of the world (Sternberg 2016). 

### 8.4. Focus Mastery and Instruction 

While the test of adaptive intelligence would provide a clear picture of what individuals know and potentially could do about global issues, the true power of a criterion-referenced test of adaptive intelligence would lie in its support of instruction. The criterion-referenced test of adaptive intelligence would not seek primarily to rank individuals or to compare their scores. It rather would seek to assess and promote growth in the individual’s vision to make positive changes in the world. For educational purposes, the test would provide standards of what learners should know, and be able to do, about global issues. Knowing the current level of adaptive intelligence of a learner would be useful to provide feedback on the learning experience and to modify instruction to enhance overall performance and underlying levels of adaptive intelligence in the population. 

The proposed kind of criterion-referenced test would directly suggest areas in which instruction would be appropriate. The instruction would be focused not just on the development of knowledge base—which of course would be important—but also on the development of creative, analytical, practical, and wise thinking for each of the world-level problems. Standards and criteria for the test could be expanded to instructional settings, establishing alignment among global priorities, education policies, and education systems (e.g., climate literacy, Stanistreet 2021). The existence of such standards also has the potential to enhance teacher preparation by outlining strategies and materials that teachers can use to enhance adaptive intelligence in school environments. 

## 9. Potential Challenges with Criterion-Referenced Assessment of Adaptive Intelligence

CRA of adaptive intelligence may face several challenges. These include:

### 9.1. Would Judgments Be Arbitrary?

Criterion-referenced testing is closely associated with the idea of standards (Glass 1978a). In this sense, a criterion-referenced assessment of adaptive intelligence would measure the attained degree of adaptive competence on a continuum marked by prespecified standards. This approach rests on the common notion that a minimally acceptable or agreed-upon level of performance on an adaptive intelligence task can be specified. 

A possible criticism of such efforts is that the criterion levels, or standards, would be somewhat arbitrary. The determination of standards, therefore, might rest on spurious and misleading claims of precision and rationality (Glass 1978a). Although this potential criticism about non-precision has some merit, some scholars (e.g., Popham 1978; Scriven 1978) have argued that human beings can arrive at consistent and nonarbitrary judgments. Therefore, selectors of criteria can set sophisticated criteria or standards when they comprehend the nature of their task, and have access to information relevant to that task. We concur with this view and therefore suggest a collaborative effort among measurement experts, educators, and content experts, to arrive at relatively precise criteria for the assessment of adaptive intelligence.

Some scholars (e.g., Norris 1991) argue that the arbitrariness in criterion selection develops in part because “like theories, standards are always going to be empirically under-determined” (p. 336). Therefore, a substantial challenge may arise, in particular, when the standards are not empirically determined at all. An allied challenge is that standards, once set, might rapidly become obsolete with the fast pace of socio-economic changes (Norris 1991). To counter this ever-present change, we suggest not relying purely on conventional or intuitive thinking about adaptive intelligence, and rather taking an empirical approach, such as the one we described above to choose the most important issues before humankind today. Of course, these issues may change with time, and so should the criteria to measure adaptive intelligence. The issues we have selected above come from today’s global priorities. Potential global catastrophic risks of the future may arise from the creation of destructive artificial intelligence, biotechnology, nanotechnology, insufficient or malign global governance, cyberterrorism, nuclear holocaust, bioterrorism (genetically modified organisms), a failure to manage a pandemic, irreversible global warming, and human overpopulation. These risks cannot be fully predicted today, and therefore, the criteria of the assessment of adaptive intelligence must themselves be adaptive to the changes occurring with time.

### 9.2. Minimal Competence

Another potential criticism of criterion-referenced testing of adaptive intelligence might be that it is impossible to pinpoint the absolute minimum level of competence that an individual needs to effectively adapt to the world and contribute to the common good (see Glass 1978a, for a discussion of this general issue). Popham (1978) offered a solution to this problem by providing an alternate view of the idea of minimal competence, that is, the lowest acceptable performance. That is, although it may not be possible to identify the absolute minimum level of adaptive competence, criterion-setters can decide on the lowest level of adaptive proficiency they consider acceptable for the set of situations at hand. For example, excessive use of natural resources can be unanimously considered as unacceptable performance; in contrast, any conscious and meaningful effort to conserve natural resources, even to a small extent (e.g., judicious use of electricity, or walking to a small-distance place), could be identified as the lowest acceptable performance on the scale of adaptive intelligence. With this approach to identifying minimal competence, it seems possible to create a sensible criterion-referenced test of adaptive intelligence.

### 9.3. Cultural Bias

Yet another challenge we envision in developing a criterion-referenced test of adaptive intelligence is that of inadvertently introducing cultural bias. Norm-referenced tests of intelligence are known to be prone to cultural bias (e.g., Gentry et al. 2021), especially verbal tests and even non-verbal tests (McCallum 2017). Similarly, the possibility of cultural bias cannot be avoided in the criterion-referenced assessment of adaptive intelligence (cf. Drew 1973). The effect of culture may impact the ways in which individuals in society think about adaptive intelligence and the ways criterion-setters measure the construct (McCallum et al. 2001; Valencia and Rankin 1985). The issues of who determines criteria and what those criteria include are paramount (Drew 1973). Even the seemingly objective and empirical selection of global priorities we have described above possibly reflects biases of the Global North, because our sources include the United Nations and top news outlets in the United States. Developers of a criterion-referenced adaptive intelligence test must address these epistemological and cultural issues sufficiently to meet the challenge of a multicultural assessment.

### 9.4. Normative Data

Typically, data generated from criterion-referenced tests, unlike norm-referenced tests, are not used primarily to compare an individual’s performance with the performance of others. That is, one may not be able to use the criterion-referenced test of adaptive intelligence to compare a test-taker’s score against another test-taker’s score, the way intelligence tests are typically used. Some critiques (e.g., Glass 1978a) have described this as a limitation of criterion-referenced testing. However, there are two caveats here. 

First, a criterion-referenced test of adaptive intelligence must be developed by creating precise descriptions of adaptive intelligence (cf. Hambleton et al. 1978). Active, normative performance data from test-takers can be effectively used to set meaningful standards (Glass 1978b; Popham 1978). In fact, an earlier critic of this approach, Glass (1978a), later went on to describe active performance data from test-takers as the best way for setting adequate standards. That is, performance data from test-takers could be used to set sensible standards to measure adaptive intelligence. For example, normatively, performance data could be used to set age-appropriate standards of adaptive intelligence.

Second, we view intelligence as malleable, that is, one can develop adaptive intelligence with training and practice. Therefore, teachers, students, and parents can benefit from the test data if they provide feedback and guidance for enhancing an individual’s adaptive intelligence. Data generated from the use of a criterion-referenced test of adaptive intelligence can help in this regard. That is, it matters less if the data the test provides are normative, or not. What matters more is that test-takers and their teachers can use the test data to develop adaptive intelligence, which would possibly help in raising overall adaptive intelligence on a large scale, beginning at an individual level. Moreover, a growth mindset of intelligence can affect people’s motivation and ultimately their accomplishments (Dweck 2017; Dweck and Yeager 2020; Walton and Wilson 2018). Therefore, a criterion-referenced assessment of adaptive intelligence potentially could promote a growth mindset of intelligence, which can lead to positive motivational and achievement outcomes. 

By combining the two caveats, that is, using active, normative performance data to set sensible standards, and using an individual’s performance data for educational purposes, both types of scores, norm- and criterion-referenced, could be important for different purposes. This combination could amplify the strength of the criterion-referenced approach to assessing adaptive intelligence.

### 9.5. Teaching to the Test

Finally, one criticism of criterion-referenced testing is that it is possible to teach to the test. Although this criticism also applies to norm-referenced testing, as evidenced by widespread SAT and ACT coaching programs, the advantage of criterion-referenced testing is the opposite; it is clearer what you are teaching to, and that clarity can be a potentially positive outcome—no more guessing around. The test-takers would know exactly what they are being assessed on, and could prepare to perform better on a test of adaptive intelligence. Doing so would possibly result in positive outcomes for the individuals, as well as for society as a whole. As the problems presented on a test of adaptive intelligence would be real-world problems, the better the test-takers are at solving the problems, the better for them, and the world.

## 10. What Might a Test of Adaptive Intelligence Look Like?

Together with collaborators, we are currently developing tests of adaptive intelligence. There is no past “formula” on which we can draw in the development of these tests. Here is an example of a problem:

“Global warming and climate change—often used interchangeably, although they have slightly different meanings—are among the most discussed issues today in the world’s political and scientific communities. Many people are concerned about the seemingly irreconcilable options of preserving life’s modern conveniences and prioritizing earth’s natural resources. On the political end of the spectrum, both activists and skeptics are fighting for their voices to be heard. The concern is persistently discussed among youths who are looking out for their own futures, and those of future generations. In terms of how much certain individuals care about this issue, there are splits by age, political ideology, education, nationality, and geography. It is no surprise that global warming remains one of the most polarizing issues in the world today.

Your task is to take this issue—global warming and climate change—and based on your understanding, prepare statements that enforce your stance and counter competing arguments.

Do your best to consider multiple perspectives on this issue. Even if you are confident in your stance, it can be extremely valuable to consider where others are coming from, so that you can argue your key points most effectively. 

Question: What are the key things that the world needs to be doing now, to deal with global climate change? Why?”

There is no right or wrong set of answers. Rather, a good answer would take a subset of steps toward reducing climate change, and stating why they would help. For example, a respondent might say something like: “There are several steps people can take right now to reduce global climate change. Although no one step by any one person is likely to have much of an impact, if everyone worked together for a common good, the effects could be substantial in reducing climate change. First, people could reduce their carbon footprint by, where possible, heating their home through solar energy by installing solar panels, buying, and using electric cars, and using bicycles whenever possible rather than hydrocarbon-fueled vehicles. Second, people could heat their homes to a lesser temperature, air condition (where air conditioning is used) to a higher temperature, and wash clothes in cooler or even cold water. Third, people could recycle more goods, avoiding the temptation just to throw things in the garbage because it is sometimes easier to do. Fourth, people could insulate their homes better and be sure to patch any holes or obvious sources of leaks from the outside. Fifth, if they have lawns, they should stop watering them and they should conserve water wherever possible. Sixth, if employers let people work from home, even one day per week, the lessening of motor-vehicle travel would help reduce the world’s carbon footprint. In general, what people need to do will vary as a function of where they live and of the circumstances in which they live. It has become clear that governments, at least at this time, are not going to do all that can be done to reduce global climate change, so everyone must take it upon themselves to do, collectively, what will help so that many small effects will add up to a large effect. Waiting for governments or companies or large entities to do all the work will not cut it at this time. People all must do their part”.

Scoring is by expert judges’ ratings of the creativity, analytic strength, practicality, and wisdom of this and other responses, in utilizing the information in the problem plus relevant prior knowledge, in terms of the criterion of reducing climate change.

We do not expect our participants to be experts on global climate change. We do expect them to be adaptively intelligent, to have some knowledge of the issue, and to display ability in understanding the problem and formulating solutions to the problem. Our scoring is with respect to knowledge base, but also creative, analytical, practical, and wisdom-based deployment of the knowledge they have (with wisdom-based deployment referring to seeking a common good; by balancing one’s own, others’, and larger interests; over the long- as well as short-term; through the infusion of positive ethical values; in order to adapt to, shape, and select environments). What concrete, valid, and executable suggestions can participants make for dealing with climate change? Although the participants’ knowledge base could be expected to be limited for any one particular problem, over a range of problems, participants could show their understanding of current world problems and options for dealing with them. This is knowledge and understanding that, in the Anthropocene era, it is important for us all to have. In this way, we can be part of the solutions, rather than of the problems, facing the world today and in the future.

It might seem like problems such as global climate change, water and air pollution, coping with violence, and stopping the spread of disease are merely somewhat arbitrary domains of specialized knowledge. We have a different point of view. We have chosen domains that are, according to the United Nations as well as other sources, representative of the most pressing problems facing humanity and its future. These are problems that face everyone, not just those who happened to study one particular domain of knowledge or another. From the standpoint of adaptive intelligence, these are problems that we all need to solve collectively. In scoring, we are not looking for an advanced knowledge base, but rather well-reasoned answers that reflect understanding of the importance of the problem, and of some of the steps all of us, not just experts, need to take in order to create a survivable world. Leaving it to the politicians has been tried—it has worked poorly. Politicians are too compromised, or perhaps too unfocused, to solve these problems that face the world today, and that require solutions. So, we are studying problems that experts have designated as demanding solutions for a sustainable and livable world, not only for our descendants, but for ourselves.

These are problems that face everyone, not just those with a particular education or who were educated at a particular time. Much, if not most of the education one gets in preparing to solve these problems, is not attained in school. Indeed, in some ideological pockets of the U.S. and other countries, teachers will be discouraged from teaching about these issues. These are issues that one needs to reason through, regardless of where or when or for how long one has gone to school. They are not problems for just the well-educated or the educated in particular specialties—they are problems for everyone.

The issues on which we survey participants are a subset of those that might be surveyed in an assessment of wisdom. Indeed, our scoring in terms of a common good; by balancing one’s own, with others’, with larger interests; over the long as well as the short term; through the infusion of positive ethical values; to adapt to, shape, and select environments derives from the balance theory of wisdom (Sternberg 2019b). In a sense, adaptive intelligence is a subset of wisdom, and it is conceptualized in terms of creative, analytically strong, practically strong, and wise solutions to problems. However, not all wisdom-based problems are adaptive-intelligence problems. Wisdom is a far larger category (Sternberg and Glück 2019). Adaptive intelligence problems are those that matter for the future of the world, at an individual, collective, and global level. Wisdom problems, according to the balance theory (Sternberg 1998), are any problems that require balancing of interests, which would mean any problems requiring conflict resolution or judgments of human relations, regardless of their relevance to the problems facing the world as a whole.

## 11. Conclusions

The approach we have taken in this article is only one of several that could be taken to study intelligence as adaptation in a criterion-referenced or related way. For example, Rasch-scaled measurement can provide scores that are norm-independent and that are invariant with respect to given item content (e.g., Stemler and Naples 2021). More generally, the field of assessment is moving toward theory-based assessments that could serve as bases of CRT and other innovations (Birney et al. 2022; Broers 2021; Kellen et al. 2021). Whatever theory is used probably needs to consider that adaptive intelligence is multidimensional, at the very least with regard to the dimensions that underlie even narrowly defined intelligence (Carroll 1993), creativity, and wisdom (Sternberg and Glück 2019).

Whatever theory is used also needs to consider that individual and group interests often differ, especially when grouping becomes tribal. Our argument is that the best reconciliation is a broad common good that transcends both individual and tribal interests.

Adaptive intelligence is essential to human existence, but no fully developed adaptive intelligence assessment exists today. Assessments play an important role in education, especially because what gets assessed gets addressed in education. In this regard, the importance of an adaptive intelligence assessment cannot be overstated. In this article, we have discussed the possibility of developing a criterion-referenced assessment of adaptive intelligence to address the challenges facing the world in the Anthropocene era. 

As uses of adaptive intelligence seek to improve the world and work toward a common good, we identified five top global priorities that the test could serve. Foreseeably, criterion-referenced testing of adaptive intelligence has several challenges and limitations. However, each of those could be possibly overcome and used to advantage. Ultimately, it might be possible to develop such an intelligence test that not only benefits an individual, but also other people, society, and the planet in the era of the Anthropocene. 

## Data Availability

There are no new empirical data reported in this article.
